# The Promoter Analysis of *VvPR1* Gene: A Candidate Gene Identified through Transcriptional Profiling of Methyl Jasmonate Treated Grapevine (*Vitis vinifera* L.)

**DOI:** 10.3390/plants11121540

**Published:** 2022-06-09

**Authors:** Faiz Ur Rahman, Ying Zhang, Irshad Ahmad Khan, Ruitao Liu, Lei Sun, Yandi Wu, Jianfu Jiang, Xiucai Fan, Chonghuai Liu

**Affiliations:** Zhengzhou Fruit Research Institute, Chinese Academy of Agricultural Sciences, Zhengzhou 450009, China; rfaiz36@yahoo.com (F.U.R.); khan.gscaas@gmail.com (I.A.K.); liuruitao10abc@163.com (R.L.); sunlei01@caas.cn (L.S.); 82101181116@caas.cn (Y.W.); jiangjiangfu@caas.cn (J.J.); fanxiucai@caas.cn (X.F.)

**Keywords:** grape, MeJA, Transcriptome, PR1, GUS, β-glucuronidase

## Abstract

Methyl jasmonate (MeJA) plays a vital role in plant disease resistance and also induces the expression of disease resistance genes in plants. In this study, a transcriptome analysis was performed on grapevine leaves after 12, 24 and 48 h of MeJA-100 μM treatment. A total of 1242 differentially expressed genes (DEGs) were identified from the transcriptome data, and the analysis of the DEGs showed that genes related to phytohormone signal transduction, jasmonic acid-mediated defense, Mitogen-activated protein kinase (MAPK), and flavonoid biosynthetic pathways were upregulated. As Pathogenesis-related gene 1 (PR1) is an important marker gene in plant defense also upregulated by MeJA treatment in RNA-seq data, the *VvPR1* gene was selected for a promoter analysis with β-glucuronidase (GUS) through transient expression in tobacco leaves against abiotic stress. The results showed that the region from −1837 bp to −558 bp of the *VvPR1* promoter is the key region in response to hormone and wound stress. In this study, we extended the available knowledge about induced defense by MeJA in a grapevine species that is susceptible to different diseases and identified the molecular mechanisms by which this defense might be mediated.

## 1. Introduction

The grapevine (*Vitis vinifera* L.) is economically a major fruit crop grown worldwide [1]. Due to global economic importance and adaptation to the diverse climatic conditions, grapevine has appeared as a model perennial fruit crop species. Generally, grapevine cultivars are susceptible to several biotic (fungi, phytoplasma, oomycetes, bacteria, viruses, and nematodes) and abiotic (light, temperature, and water availability) factors, which dramatically effect plant growth, development, yield, and fruit quality [2]. According to their infection strategies, pathogens in plants can be divided into necrotrophics, biotrophics, and hemibiotrophics. Necrotizing pathogens extract nutrients from dead cells and during the colonization process; they secrete lyase and phytotoxin to promote cell necrosis in host plant. On the other hand, biotrophic pathogens feed on living tissues to maintain the viability of the host to obtain metabolites. At the end, hemibiotrophics pathogens start from the biotrophic infection stage to the late necrotizing infection stage [3]. 

Plant immunity consist of many mechanisms by which plants diagnose a pathogen attack, and provide the information through signaling networks within the cell, to adjacent cells and distant tissues. The early (10–30 min after pathogen attack) defense responses in plant–pathogen interactions, which are based on basal defenses in which extracellular elicited pathogen- or microbial- associated molecular patterns (PAMPs or MAMPs) are recognized by trans-membrane PRRs (pattern recognition receptors). The pathogenic components are PAMPs such as bacterial flagellin, which is a structural component of bacterial flagellum, a bacterial elongation factor Ef-Tu and fungal chitin, recognized by Flagellin-sensing 2 (FLS2), and EF-Tu receptor (EFR), respectively. In general, the PRRs results in Pathogen triggered immunity (PTI) are the recognition of PAMPs/MAMPs. The functions of PTI competently are against a wide range of microbes and PTI signaling is proliferated via Mitogen-activated protein kinase (MAPK), MAPKK, and MAPKKK cascades, which leads to phosphorylation and activates the expression of defense-related genes. The activated PTI also leads to retard growth as trade-off between growth and defense physiology [4,5]. In plant defense system, several plant hormones, particularly Jasmonic acid (JA), Salicylic acid (SA), and ethylene (ET), perform as a signal molecule that activates downstream defense reaction [6,7]. Interestingly, ET and JA are generally responsible for defense response related to herbivores and necrotrophic pathogens, while SA is responsible for defense response associated with biotrophic pathogens [8].

JA and methyl jasmonate (MeJA) in higher plant species are known as endogenous signaling molecules, because they take part in various developmental processes and stress-related conditions [9,10]. Exogenous MeJA treatment triggers specific enzymes such as chalcone synthase and phenylalanine ammonia-lyase (PAL), which are involved in plant defense responses and also produce compounds that are related to plant defense, such as polyphenols, alkaloids, or pathogenesis-related (PR) proteins [11,12]. MeJA have the ability to protect the plants from various diseases caused by biotrophic and hemibiotrophic pathogens by inducing a systemic resistance in Arabidopsis, maize, and wheat [13,14,15]. In a couple of decades, various genes and transcription factors (TFs) related to JA biosynthesis and signal transduction process have been identified in response to different environmental signaling [16]. For example, JASMONATE ZIM-DOMAIN (JAZ) proteins overwhelm the expression of the JA-receptive gene through interaction with the MYB and MYC TFs [17]. Similarly, NAC, MYB, ERF, and WRKY TFs exhibit a notable response to JA signaling. Additionally, in *Arabidopsis*, numerous WRKY genes, which are the activator of pathogenesis related proteins, such as WRKY22 [18], WRKY57 [19] WRKY50 [20], and WRKY70 [21], are mainly linked with plant defense purposes, which are controlled by the JA signaling pathway. 

Next-generation sequencing (NGS) techniques are modern techniques to identify the changing cellular transcriptome in the biological samples [22,23]. In animals, transcriptome study was used widely; however, in plants, it was not common, but these days, this technique has also been extensively used [24,25]. In the past few years, MeJA has been widely used in strawberries [26], peaches [27], apples [28], and mangoes [29]. Recently, the application of MeJA in grapevine has gained more and more attention [30], which decreases the potential attack of postharvest diseases in grape berries. However, to date, no transcriptomics study has been reported on the exogenous application of MeJA on the grapevine leaves against biotic or abiotic stress. Therefore, the present study was performed to investigate the MeJA responses on a susceptible grapevine cultivar through RNA sequencing to identify the potential candidate genes responsible for the defense responses. For further functional analysis, promoter of the *VvPR1* gene, one of the candidate genes, was isolated and serially deleted into different fragments for the transient expression in tobacco leaves by a GUS reporter gene to identify the cis-regulatory elements responsible for the activation of *VvPR1* genes in response to MeJA treatment.

## 2. Results

### 2.1. Transcriptomic Analysis of Grapevine Leaves in Response to MeJA at Different Time Points

The grapevine leaves sprayed with MeJA treatment were examined for transcriptomics changes after 12, 24, and 48 h of treatment. As the most important diseases attack the aerial parts of plants, grapes leaves were selected for the MeJA treatment. A total of 76.5 Gb data of clean reads were collected from the 12 leaf samples, and individual leaf sample contained ≥6.3 Gb data with a Q20 quality score of ≥97.1% (Table 1). In addition, for each leaf sample, more than 86.62% reads were uniquely mapped. The reads aligned with the reference genome of the *V. vinifera* L., and MeJA-treated samples were compared with the control. 

**Figure 1:**
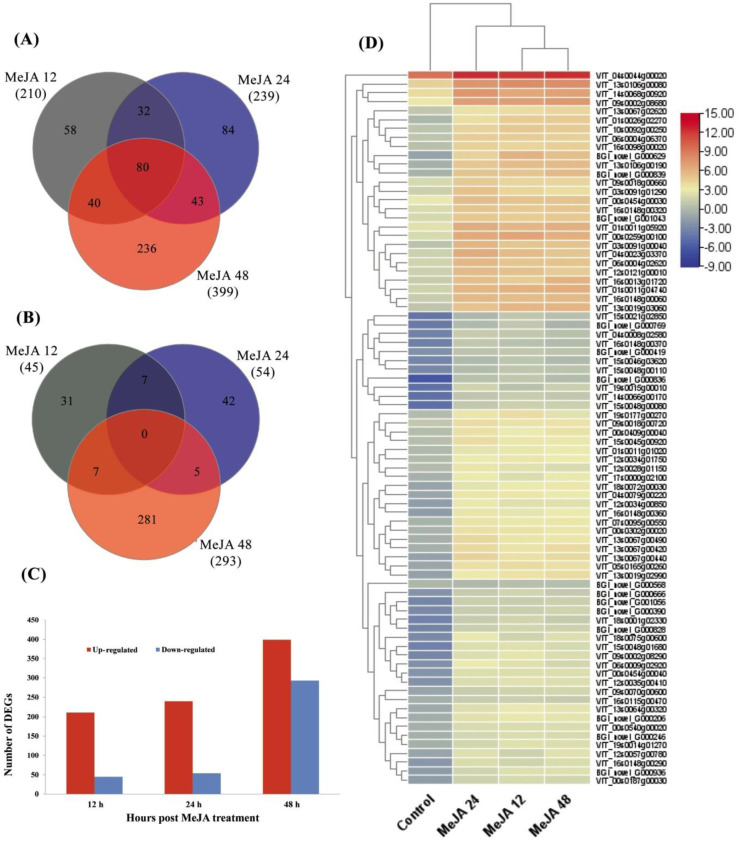
Overview of RNA-seq data with the application of MeJA at different time courses. (**A**) Venn diagram elucidating the number of genes upregulated by MeJA treatment over the different time points. (**B**) Venn diagram elucidating the number of genes downregulated by MeJA treatment over the different time points. (**C**) Total number of transcripts that was significantly up- or downregulated in response to MeJA treatment. Log2 FC ≥ 1 or ≤ −1 and *p* < 0.01 FDR. (**D**) Heatmap of common upregulated genes found at all time points.

After the MeJA treatment, a large number of transcripts changed their expression levels (*p* ≤ 0.05) on the basis of the Cuffdiff analysis. The Log_2_-fold change (Log_2_FC) ≥ 1 or ≤ −1 and *p*-value ≤ 0.05 for DEGs were used as the threshold values with respect to different time points. A total 1242 DEGs were found from the grapevine leaf samples after MeJA treatment at different time points (12, 24, and 48 h). To calculate and represent the distribution of up and downregulated genes at different time points, a Venn diagram was used. Among the upregulated, a total of 378 genes were unique set of genes at all three time points, and there were 80 coregulated genes at all time points induced by MeJA (Figure 1A). Moreover, among the downregulated genes, a total of 354 genes were unique genes out of which 281 were identified at 48 h, and there was no co-repressed gene at all time points (Figure 1B). Briefly, 256 (211 up- and 45 downregulated), 294 (240 up- and 54 downregulated), and 692 (399 up- and 293 downregulated) DEGs were identified at 12, 24, and 48 h respectively (Figure 1C). DEGs common in the upregulated Venn diagram at all time points after the application of MeJA are shown in a heatmap (Figure 1D).

### 2.2. Gene Ontology (GO) Analysis of DEGs

For the functional classification of DEGs, GO analysis was performed of the transcriptomics data from the grapevine leaves on the exogenous application of MeJA. GO functions were characterized in the three main groups: biological process, cellular component, and molecular function. The group of biological process consisted of 16 GO terms, the cellular component group consisted of 3 GO terms, and the group molecular function consisted of 10 GO terms (Table 2). At all time points, in the category of biological process, the upregulated DEGs were enriched in response to stimulus, cellular process, and metabolic process. In the category of the cellular component, a large proportion of DEGs (upregulated) induced by the MeJA were enriched in cellular anatomical entity and intracellular. In the molecular function category of GO, a large proportion of DEGs (upregulated) induced by the MeJA were enriched in catalytic activity.

From all the time points after the MeJA treatments, the most upregulated DEGs were enriched in the biological process category at MeJA-48, which comprised of a cellular process (38.4%) and metabolic process (18.3%). In the category of the cellular component, most upregulated DEGs were found in an anatomical (61.8%) entity and intracellular (30.5%), and in the molecular function category, most upregulated genes were identified in binding (46.5%) and catalytic activity (44.5%) (Table 2). From all the time points, in the category of biological process, the downregulated DEGs were significantly found in metabolic processes and cellular processes at MeJA-48. In the cellular component category, the downregulated DEGs were significantly found in the cellular anatomical entity. Moreover, in the category of molecular function, more downregulated DEGs were found in the catalytic activity at all the time points, further indicating the reduction of catalytic activities (Table 2).

### 2.3. Kyoto Encyclopedia of Genes and Genomes (KEGG) Analysis of DEGs

**Figure 2:**
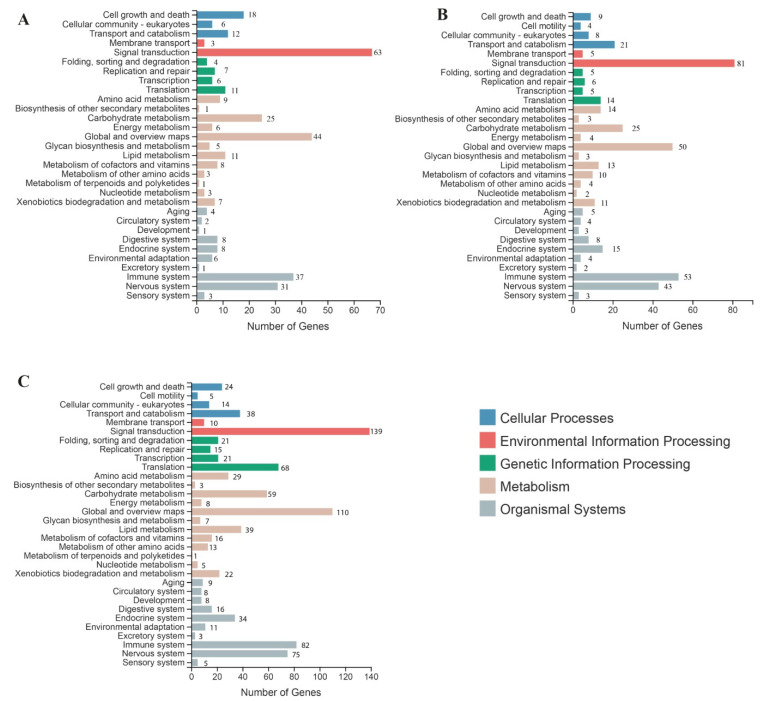
Kyoto Encyclopedia of Genes and Genomes (KEGG) analysis of specifically differentially expressed genes (DEGs) at 12, 24, and 48 h of MeJA treatment: (**A**) 12 h-post treatment, (**B**) 24 h-post treatment, and (**C**) 48 h-post treatment.

To explore the functional networks and biological pathways, a KEGG enrichment analysis was performed. A total of 160 KEGG pathways contained 692 DEGs from all the DEGs. The KEGG metabolic pathway divided into five branches: Cellular Processes, Genetic Information Processing, Environmental Information Processing, Organic Systems, and Metabolism. In this grapevine transcriptome analysis, the most enriched KEGG pathways were signal transduction (63, 81, and 139); global and overview map (44, 50, and 110); immune system (37, 53, and 88); translation (11. 14, and 68); and carbohydrate metabolism (25, 25, and 59) at 12 h, 24 h, and 48 h treatment, respectively (Figure 2A–C). From all the KEGG pathways, the top 20 were defense- and immune-related pathways, among them the MAPK signaling pathway, Toll-like receptor signaling pathway, and Ras signaling pathway, of the plant, and activation of defense related genes have crucial role in disease resistance of plants (Supplementary Figure S1).

### 2.4. Transcript Levels of Genes Involved in JA Dependent Defense Pathway in Grapevine Leaves after MeJA Treatment

The Jasmonic acid pathway is commonly involved in defense response against different types of pathogens and pests. According to the transcriptome data in Table 3 and Supplementary Table S3, PLD involved in the JA metabolism was upregulated (1.13–1.57-fold) and (6.73–17.51 FPKM) of MeJA treatment at all time points. Two lipoxygenase (LOX) genes were significantly upregulated after MeJA treatment (0.27–3.11-fold) and (0.03–17.59). Two ACX genes were significantly upregulated (1.57–3.35-fold) and (0.16–20.32 FPKM) after MeJA treatment and one phenylalanine ammonia-lyase (PAL) gene was also upregulated (2.46–3.27-fold) and (3.48–53.44 FPKM). Two chalcone synthase (CHS) genes were also identified from the DEGs, and both were significantly upregulated (1.70–2.98-fold) and (15.78–380.29) at all time points except one, which is not expressed at 12 h of MeJA treatment. Two (F3H) genes were also identified from the DEGs, both were significantly upregulated (2.18–2.99-fold) and 2.88–140.48 FPKM, after MeJA treatment. MYB, WRKY, PR1, PR4, and bHLH genes were also involved in the metabolism of JA, two MYB (0.12–2.59-fold) and (2.71–156.89), two WRKY (0.21–0.98-fold) and (12.47–29.44 FPKM) genes were also significantly upregulated after MeJA treatment. Pathogenesis-related gene 1 (PR1) and PR4 were also significantly induced (0.08–2.49-fold) and (51.50–7316.73) in response to MeJA, and one bHLH was also significantly induced (0.40–1.20-fold) and (17.40–37.55 FPKM) (Table 3 and Supplementary Table S3).

### 2.5. Mitogen-Activated Protein Kinase (MAPK) Pathway

**Figure 3:**
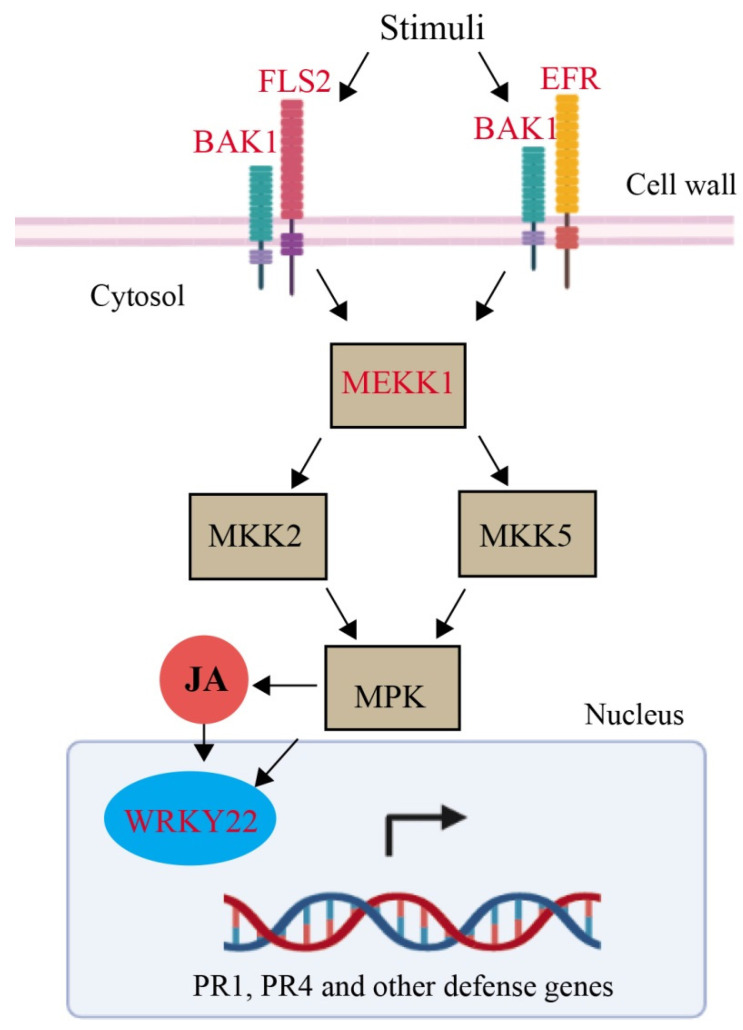
MAPK-WRKY pathways in response to MeJA. The diagram of MAPK-WRKY pathways. The abbreviations for the enzymes; Brassinosteroid (BR) insensitive1 (BRI1), FLAGELLIN-SENSITIVE 2, BRI1-associated kinase 1 (BAK1) (FLS2), Mitogen-activated protein kinase kinase kinsase (MEkK1), Pathogenesis related gene-1.

Brassinosteroid (BR) insensitive1 (BRI1), was only considered as co-receptor of brassinosteroid (BR) regulate the plant development but now BRI1-associated kinase 1 (BAK1) play a critical role in resistance of various pathogens. Thus, BAK1 mediates in PAMP-triggered immunity (PTI) and BR signaling and in defense response BAK1 play an important role. Additionally, BRI1 and BAKI was significantly upregulated (0.60 to 4.98-fold) of treatment MeJA. In animal and plant the innate immunity were played by Pattern recognition receptors (PRRs). PRR binding of their cognate ligand activates an immune response and triggers a signaling network. A one of the best leucine-rich repeat (LRR) receptor kinase is FLAGELLIN-SENSITIVE 2 (FLS2) which recognizes a conserved 22-amino acid peptide (flg22) from bacterial flagellin together with co-receptor BAK1 activates the MAPKs and immunity. The expression of FLS2 induced (0.25–3.40-fold) at all the time points after the MeJA treatment, and another probable LRR receptor-like serine/threonine-protein kinase EFR was also induced (2.16–3.39-fold) (Table 4). Mitogen-activated protein kinase (MAPK) modules play important roles in the transduction of developmental and environmental signals to downstream signaling targets via phosphorylation, including other enzymes, kinases, transcription factors, or cytoskeletal proteins in all eukaryotic cells. The MAPK plays a vital role in plant immunity and also regulate the WRKY transcription factor (TF) along with the defense mechanism of plants. The transcript level of MAPK was upregulated (3.99) and at 48 h after MeJA treatment. The WRKY22 expression was also upregulated (0.41–0.97-fold) of MeJA treatment. Pathogenesis-related gene 1 (PR1) and (PR4) were also significantly induced (0.08–2.49-fold) in response to MeJA (Figure 3) and (Table 3).

### 2.6. Verification of Differential Gene Expression

**Figure 4:**
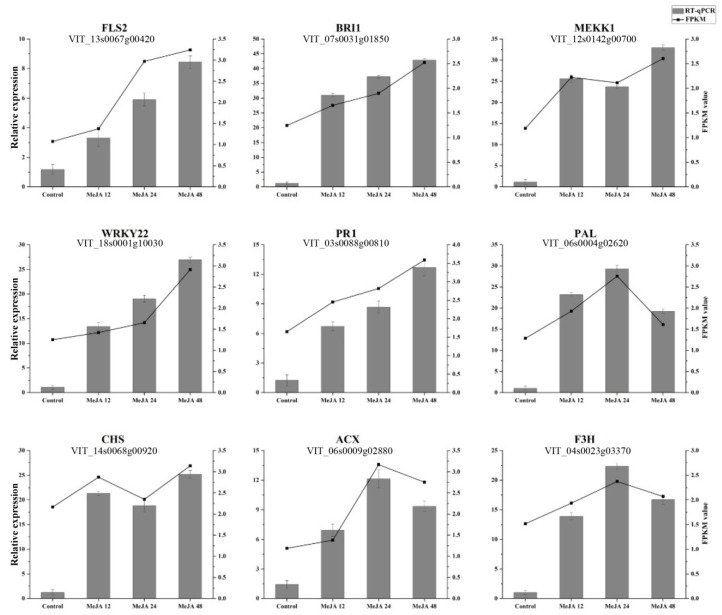
RNA-Seq data validation with RT-qPCR. Verification of RNA-Seq data with the expression level of RT-qPCR of the selected DEGs. Error bars indicate the standard error as mean + SD.

The accuracy of transcriptomic data was determined by selecting randomly genes from the DEGs. A total of nine genes were selected for qPCR verification. Four genes FLS2, BRI1, WRKY22, and PR1 were continuously increasing their expression at all time points, while the expression of MEKK1 and CHS were decreased at 24 h, whereas ACX and F3H increase their expression at 24 h. The levels of expression of all genes were found consistent with the RNA-Seq results (Figure 4). As PR1 is the most important pathogenesis-related gene in response to different biotic and abiotic stress in plants, this gene was selected for functional validation in response to MeJA treatment. 

### 2.7. VvPR1 Promoter Isolation and Sequence Analysis

**Figure 5:**
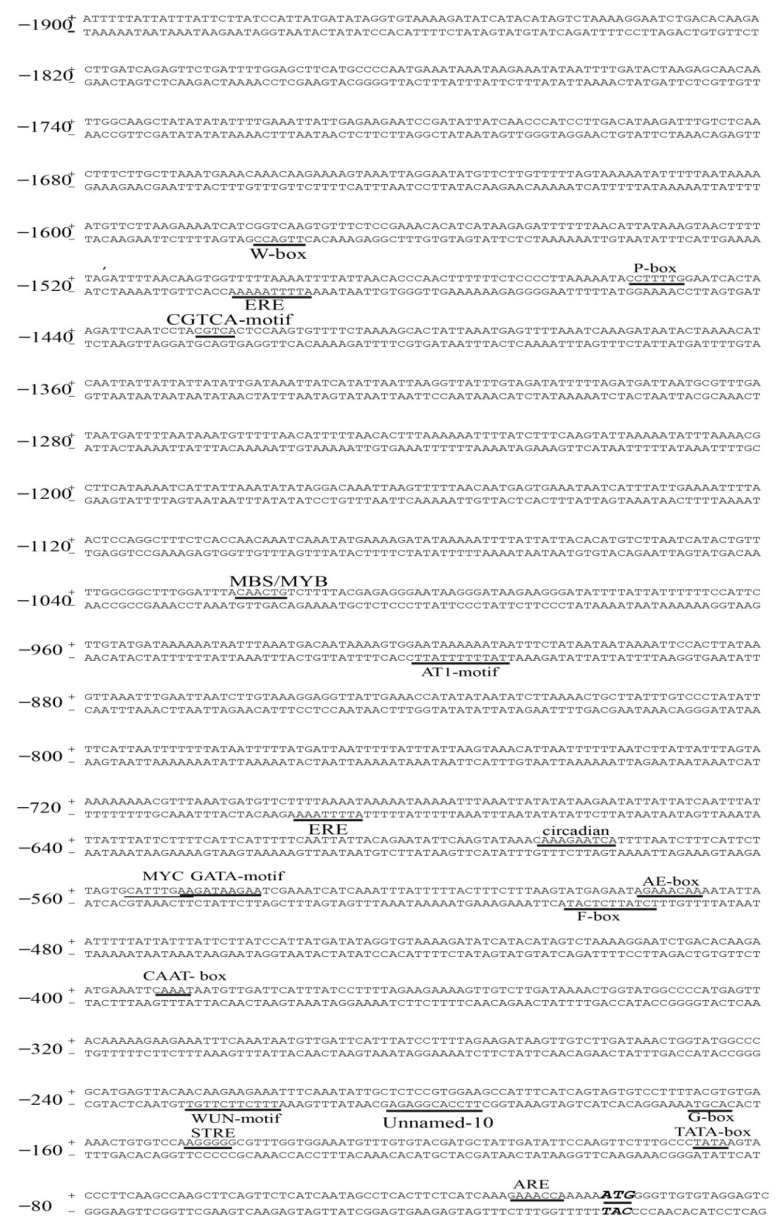
The 5′ upstream nucleotide sequence starting from the transcription start site of the *VvPR1* gene containing the putative promoter and predicted cis-acting elements. The underlined sequences showed the sequence of putative cis-regulatory elements of the promoter. Promoter contained hormone-responsive elements (ERE, P-box, and CGTCA-motif); light-responsive elements (AE-box, AT1, G-box, and chs-CMA1a); stress-responsive elements (MBS, MYC, and ARE); and other growth-associated elements (O2-site and circadian) that involved in zein metabolism regulation and circadian control. F-box and Unnamed_10 were found cis-elements with unclear function.

From the candidate genes found in DEGs, the pathogenesis-related genes (PR1 and PR4) was found one of the most important marker genes in the disease resistance against the biotrophic and necrotrophic pathogens. As *VvPR1* is less-studied compared to the MeJA treatment, *VvPR1* was selected for further validation of RNA-seq data, in which the putative promoter of an upstream region about 1900 bp from the *VvPR1* gene was isolated and cloned in a *pCE2* Blunt vector. Sequence analysis was performed by the PlantCARE database (http://bioinformatics.psb.ugent.be/webtools/plantcare/html/) (accessed on 17 February 2022) that exposed several cis-acting regulatory elements and motifs sequences that were found in the promoter of many genes in eukaryotic organisms for gene regulation and expression (Figure 5 and Supplementary Table S4). Potential regulatory elements related to stress, defense, and hormones found in other plant promoters were also found in the *VvPR1* promoter. The *VvPR1* promoter enriched with TATA-box and CAAT-Box: (a) hormone-responsive elements (ABRE, ERE, TCA-element, P-box, and CGTCA-motif); that are responsible for ABA, ethylene, SA, gibberellic acid, and MeJA; (b) light-responsive elements (AE-box, AT1, G-box and Box 4, LS7, and chs-CMA1a); (c) stress-responsive elements (MBS, LTR, and ARE) confer responsiveness to drought low temperature and anaerobic conditions, and (d) other growth-associated elements (O2-site and circadian) that involved in zein metabolism regulation and circadian control. F-box and Unnamed-10 were found cis-acting elements with unclear function. 

### 2.8. Deletion Analysis of the VvPR1 Promoter against MeJA Treatment

**Figure 6:**
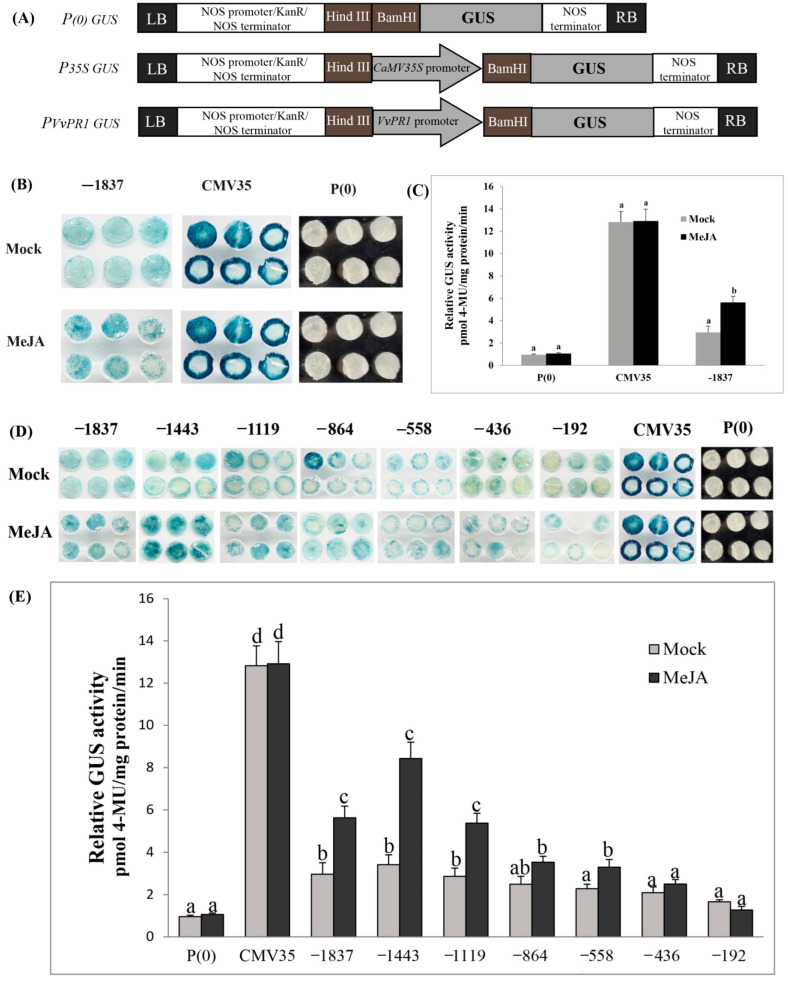
(**A**) Scheme of GUS vector construction. (**B**) Histochemical staining analysis of the full-length promoter of *VvPR1* Gene with the positive and negative control. (**C**) Fluorometric analysis of the full-length promoter of the *VvPR1* gene with positive and negative control. (**D**) Histochemical staining analysis; transiently transformed GUS expression in *N. benthamiana* leaves with serially deleted *VvPR1* promoter fragments (−1837, −1443, −1119, −845, −558, −436, and −192 pb) against different phytohormones methyl jasmonate (MeJA). (**E**) Fluorometric analysis; transiently transformed GUS expression in *N. benthamiana* leaves with serially deleted *VvPR1* promoter fragments (−1837, −1443, −1119, −845, −558, −436, and −192 pb) against MeJA. The different letters on bars in this figure represents a significant difference according to the least significant difference (LSD) (*p* < 0.05) in serially deleted *VvPR1* promoter fragments against MeJA treatments.

To explore the function of *VvPR1* promoter, the promoter sequence was cloned into the binary expression vector *pBI121*::GUS with the scheme (Figure 6A). Fluorometric and histochemical staining assays were performed to examine the activity of GUS proteins in tobacco (*N. benthamiana*) plants. The transient expression of *VvPR1* promoter showed the activation of GUS protein after the MeJA treatment on the tobacco leaves. The GUS activity in the positive control (*P35S*::GUS) was highest at about 4.30-fold, followed by MeJA treatment (2.58-fold) on the full-length promoter with respect to the mock (Figure 6B,C). To further explore the *VvPR1* promoter, the promoter sequence was serially divided into seven fragments upstream from the translational start site (−1837 bp, −1443 bp, −1119 bp, −864 bp, −558 bp, −436 bp, and −192 bp) and cloned into the expression vector *pBI121*::GUS (Figure 6A). The treatment of MeJA on serially deleted promoter fragments of *VvPR1* showed the activation of GUS protein in tobacco leaves. The GUS activity in the positive control (*P35S*::GUS) was highest about 12.91-fold with respect to the negative control *P(0)*, and significantly, there was no difference between the mock and MeJA treatments. GUS activity was significantly induced with MeJA treatment between the promoter fragments of −1837 bp to −588 bp (Figure 6D,E). The *VvPR1* promoter fragments −1837 bp and −1443 bp induced the highest GUS activity. The −1119 bp promoter fragment of *VvPR1* showed intermediate GUS activity, and the remaining promoter fragments −436 bp and −192 bp were not significantly induced with respect to the mock.

### 2.9. Deletion Analysis of the VvPR1 Promoter against Wound Stress

**Figure 7:**
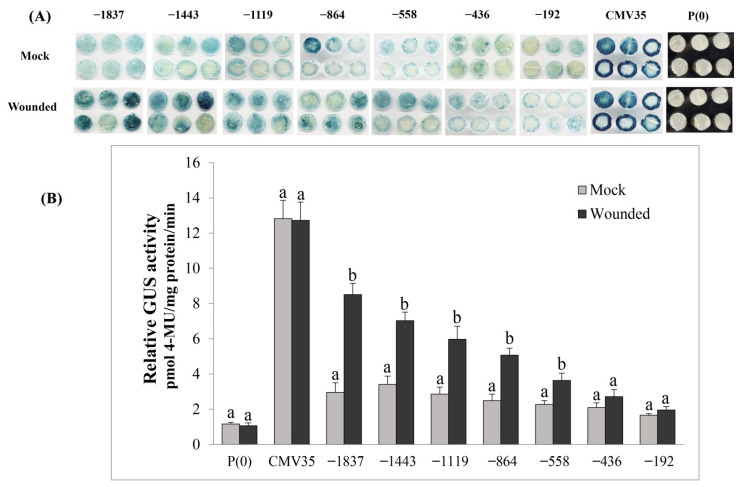
(**A**) Histochemical staining analysis; GUS activity in transiently transformed *N.* benthamiana leaves with serially deleted *VvPR1* promoter fragments (−1837, −1443, −1119, −845, −558, −436, and −192 pb) against wound stress. (**B**) *VvPR1* promoter expression in response to wounding transiently transformed with the GUS reporter gene fused with *VvPR1* promoter fragments in tobacco leaves. Different letters on the bars showed a significant difference according to the least significant difference (LSD) test (*p* < 0.05).

The effect of the wound on the activation of the *VvPR1* promoter was explored by GUS activity assay in tobacco leaves harboring promoter-GUS chimeric constructs. The *VvPR1* promoter was examined by a series of deletion fragments upstream from the transcription start site. The *VvPR1* promoter fragments from −1837 bp to −558 bp significantly triggered the GUS expression with respect to the mock (Figure 7A,B). The −1837 bp had shown the highest GUS protein activity followed by a gradually decrease in the GUS activity up to −558 bp promoter fragment. Other *VvPR1* promoter fragments from −558 bp to −192 bp did not show a significantly increase in GUS protein activation with respect to the mock.

## 3. Discussion

The current study was designed to explore the molecular responses of grapevine leaves on the exogenous application of MeJA. Transcriptomics data revealed that total 1242 DEGs were identified (either downregulated or upregulated) at all time points (12 h, 24 h, and 48 h post treatment). A number of genes related to the lipid metabolism, such as fatty acid biosynthesis/degradation, biosynthesis of unsaturated fatty acids, linoleic acid metabolism, and alpha-linolenic acid metabolism, showed differential expression in grapevine leaves after MeJA treatment. The DEGs associated with alpha-linolinic acid metabolism were found in the transcripts that play an important in JA biosynthesis, including one PLD, two LOX (lipoxygenase), and ACX (Table 3). These genes were found in the JA biosynthesis pathway. In response to the environmental factors jasmonates regulate the transcriptional changes, which induce the defense genes and the unsaturated fatty acid alpha-linolenic acid is conserved in jasmonates production [31]. This result suggests that the positive regulatory feedback mechanism which is necessary for the JA biosynthesis is present in the study. In many crop plants such as tobacco and tomato reported that the exogenous MeJA application induced the expression of genes related to JA signaling pathway and JA biosynthetic enzymes [32,33,34]. We hypothesized that transcription factors regulate the genes expression related to the cellular functions in response to MeJA. Some important transcription factors for the JA responsive genes were also differentially expressed in the grapevine samples such as MYB, WRKY, and bHLH in response to the MeJA treatment. These transcription factor families were previously exposed as transcriptional regulators of plant defense genes against jasmonate [35,36,37]. Phenylpropanoids are a group amino acid of aromatic, among the plant secondary metabolites; physiologically active secondary metabolites which are derived from phenylalanine such as isoflavonoids and anthocyanins [38]. Phenylpropanoids play a vital role abiotic and biotic stimuli also contribute to aromas/scents [39]. In our DEGs, MeJA treatment influenced the phenylpropanoid biosynthesis. Phenylalanine ammonia-lyase induced by 3.26-fold, which might lead directly towards more cinnamic acid synthesis in the phenylpropanoid biosynthesis pathway. In proceeding to phenylpropanoid biosynthesis, the pathway chalcone synthase (CHS) was significantly induced, leading towards the chalcone from 4-coumaroyl-CoA. In addition, the flavanone 3-hydroxylase (F3H) gene was also significantly induced by the MeJA treatment in grapevine leaves and converted flavanones to dihydrokaempferol. The most common plant pigments are flavonoid, which takes part in plant stresses [40], and flavonoid-derived compounds were also affected by the MeJa treatment. Transcriptome analysis also revealed that MeJA elicitation affect the DEGs related to the flavonoid pathway. 

Figure 3 and Table 4 showed that the MAPK signaling pathway and other metabolic pathways were enriched in the samples of grapevine leaves after the MeJA treatment. Several MAPKs have been found to involve in plant defense response [41,42,43,44,45,46]. Genetic and biochemical approaches exposed that BAK1 is a co-receptor of BRI1, which is essential for full activation of BR signaling by physically interaction with BRI1 [47,48]. In term of functions, BAK1 also perform an important role in plant immunity to pathogens. Plantsare found compromised in their resistance to oomycete and bacterial pathogens in *N. benthamiana BAK1* mutants. In a wild tobacco (*N. attenuate*) plant, it was also found that on mechanical wound and insect feeding, BAK1 regulates JA accumulation [49]. Therefore, in various signaling pathways, BAK1 appears to be an important hub. In this study, both BRI1 and BAK1 genes were significantly upregulated after the 24 h of MeJA treatment, inducing the downstream genes related to plant defense. 

During a pathogen attack, plants depend on their innate immunity. On the base of mode of action two different types of defense systems were identified. Plants can recognize pathogen/microbe-associated molecular patterns (PAMPs/MAMPs) by PRRs and activate PAMP-triggered immunity (PTI) [50]. Moreover, R (resistance) proteins in some plants or plants species recognize pathogen-derived effectors and activate effector-triggered immunity (ETI) [51], which initiates a strong defense response, such as a hypersensitive response [52].

Five FLS2 (flagellin-sensing 2), one BAK1, and one BRI1 genes were enriched in the DEGs of grapevine leaves after the MEJA treatment. The expression of these genes increased with the time points from 12 h to 48 h, and the maximum values were evaluated at 48 h. FLS2 (flagellin-sensing 2) is well-characterized for bacterial flagellin from all the PRRs [53,54]. FLS2 makes a complex rapidly with BAK1 in a ligand bacterial flagellin (flg22) [55,56], and the phosphorylation of FLS2 and BAK1 is induced rapidly [57], which elevates the reactive oxygen species (ROS) and activates the MAPK cascades [58]. Similarly, the Mitogen-activated protein kinase kinase kinase 1 (MEKK1) gene was significantly upregulated in the samples of grapevine leaves after 48 h of MeJA. The expression of the WRKY22 transcription factor was also induced and significantly upregulated at the 48 h of MeJA treatment. The phosphorylation of MEKK1 induces the downstream genes of MAP kinase and then interacts with WRKY22 transcription factors. The presence of the WRKY transcription factor, which has been recognized as substrates of MAPKs signaling pathways, is an important component for the activation of plant immunity [59,60,61,62]. WRKY transcription factors phospho-mimicking mutants are found in the induction of RBOHB-dependent ROS burst and cell death in *N. benthamiana*, providing the evidence that WRKY-MAPK are involved in the induction of hypersensitive-response cell death [62]. In the previous study, it was found that WRKY’s family members identified their binding sites in the PR1 promoter and activate the PR1 gene expression [63]. 

In this study, the *VvPR1* promoter of -1837 bp length was also obtained from grapevine leaves. The promoter analysis revealed that CAAT-boxes and TATA-boxes were enriched in the *VvPR1* promoter; in spite of this, other cis-acting elements were also detected, such as hormone, stress, light-responsive elements, and growth and development-associated elements. TATA-boxes are found abundant in stress-related genes and are absent in essential genes [64,65]. They are linked with a variable and rapid gene regulation [66,67,68]. Furthermore, it was also studied that plant hormones also induce the PR1 gene, but their expression pattern in different plants varies by the same hormone. Previously studied, pathogens, hormones, and different stresses activate the pepper PR1 promoter, possibly by trans-activating the transcription factors CARAV1 and CAZFP1 [69]. 

The CABPR1 promoter expression was activated upon MeJA treatment during pathogen infection in the leaf tissues of pepper [69]. In this study, the MeJA exogenous application induced the GUS expression on the tobacco leaves through the *VvPR1* promoter. From all the 5′ deleted constructs, only three −1837 bp, −1443, and −1119 bp constructs were sufficient for high GUS induction upon the MeJA treatment about 1.90-, 2.47-, and 1.87-fold respectively. The strong GUS protein expression of -1443 bp promoter may indicate that there were some suppressing elements between −1443 bp and −1837, which may reduce the GUS expression of full-length promoter −1837 bp upon MeJA treatment. Furthermore, the −864 bp and −558 bp *VvPR1* promoter fragments also induced significant GUS protein expression, but the expression was low as compared to the −1443 bp. This GUS protein expression may be due to the presence of CGTCA-motif and G-box, which are cis- elements involved in the MeJA-response located at −1422 bp and −163 bp upstream from the transcription initiation start site, respectively; our results were found consistent with a previous study [69]. TGA-bZIP transcription factors recognize the TGACG-motif and perform a vital role in the regulation of basal resistance, having moderate effects on PR gene expression [70]. Previously, it was studied in JAZ gene family promoters that contain CGTCA-motif (CGTCA), G-box (CACGTG), and TGACG-box (TGACG), cis-elements are involved in GUS expression under MeJA treatment [71].

In this study, the *VvPR1* promoter induced GUS expression under wounding from full-length −1837 bp to −864 bp with a gradually decrease in expression about 2.8-, 2.4-, 2.0-, and 1.90-fold, respectively. The induction of GUS expression between −1837 and −864 may be a due presence of stress-related cis-elements like W-box, MBS MYB, and MYC. However, the gene families such as bZIP, MYB, MYC, and WRKY transcription factors could also be good candidates for identifying wound-inducible promoters [72]. 

## 4. Materials and Methods

### 4.1. Plant Material and Hormones Treatment

Two-year old grapevine (*Vitis vinifera* L. cv. Zaotianmeiguixiang) plants were grown in pots under standard greenhouse conditions (25 ± 5 °C), 65% relative humidity (RH), 16-h light/8-h dark photoperiod, and at Zhengzhou Fruit Research Institute (ZFRI), Chinese Academy of Agricultural Sciences (CAAS), Henan, China. The cultivar under this study was a commonly growing table grape cultivar, susceptible to the grape white rot disease under the pathogenesis test conducted at Zhengzhou Fruit Research Institute, CAAS (unpublished data). Potting media contained sand and peat (50:50, *v*/*v*) and watered twice in a week. MeJA (Sigma Aldrich Chemicals GmbH, Schnelldorf, Germany) dissolved in 10% ethanol with 0.2% tween-20 with the final concentration of 100 µM. The plants were sprayed with the solution until small drops were dropping from the leaves and the leaf samples were collected after 12 h, 24 h, and 48 h of MeJA treatment and 0.2% tween-20 was used as control. The grape samples were immediately stored at −80 °C. From three different plants, three leaves under the same treatment were pooled and considered as one replication for each treatment. Three replicates were considered for each treatment therefore for control, and treatment (12 h, 24 h, and 48 h); the total number of samples was 12. Tobacco (*Nicotiana benthamiana*) plants were grown in a potting media (vermiculite/perlite/moss, 2/3/5, *v/v/v*) under controlled conditions with a 16-h light/8-h dark photoperiod 25 ± 2 °C. The *N. benthamiana* plants at the 6-th leaf stage were used for the *Agrobacterium*-mediated transient assay. 

### 4.2. Total RNA Extraction

The total RNA was isolated from the plant tissue by CTAB-PBIOZOL reagent and ethanol precipitation protocol according to the recommendations mentioned in the manual. About 80 mg leaf samples were ground in liquid nitrogen to powder and 1.5 mL CTAB-pBIOZOL reagents preheated at 65 °C added in the powder. For complete nucleoprotein complexes dissociation, at 65 °C the samples were incubated for 15 min by Thermo mixer and centrifuge at 4 °C for 5 min at 12,000× *g*. In the 1.5 mL supernatant of CTAB-pBIOZOL reagent 400 µL of chloroform was added and centrifuged at 12,000× *g* at 4 °C for 10 min. In the new 2.0 mL tube the supernatant was transferred, 200 µL chloroform, 700 µL acidic phenol was also added and centrifuged 12,000× *g* at 4 °C for 10 min. The equivalent volume of chloroform in aqueous phase was mixed and centrifuged for 10 min at 4 °C at 12,000× *g*. The isopropyl alcohol with the equal volume of supernatant mixed with the supernatant and keep for precipitation at −20 °C for 2 h. Then supernatant was removed from the mixture centrifuged at 12000× *g* at 4 °C for 20 min. The RNA pellet was air-dried after washing with 1 mL of 75% ethanol in the biosafety cabinet and was dissolved with DEPC-treated water by adding 50 µL of volume. Subsequently, total RNA was quantified and qualified using a Nano Drop and Agilent 2100 bioanalyzer (Thermo Fisher Scientific, Waltham, MA, USA). 

### 4.3. Library Construction of mRNA and Data Analysis

The mRNA attached with Oligo (dT) was purified by magnetic beads. At appropriate temperature, fragment buffer was used to fragment the mRNA into small fragments. The random hexamer-primed reverse transcription was used to synthesize First-strand cDNA, then a second-strand cDNA generated. For the end repairing RNA Index Adapters and A-Tailing Mix were added in mixture. The cDNA fragments were amplified by PCR obtained from previous step, and then dissolved in EB solution after the purification by Ampure XP Beads. For quality control, on the Agilent Technologies 2100 bioanalyzer, the product was validated. To get the final library, the double stranded PCR products obtained from the last step were heated denatured and circularized by the splint oligo sequence. The final library was considered as the single strand circle DNA (ssCir DNA). DNA nanoball (DNB) were made from the final library amplified with phi29, which had than 300 copies of one molecular, DNBs were loaded into the patterned nanoarray and pair end 100 bases reads were produced on DNBSeq platform (BGI-Shenzhen, China). This project uses the filtering software SOAPnuke (v1.4.0) independently developed by BGI for filtering. First of all remove the reads containing the connector (connector contamination) and reads with unknown base N content greater than 5% were removed. The low-quality reads (we define reads with a quality value of less than 15 to account for more than 20% of the total number of bases in the reads as low-quality reads) were also removed. The filtered “Clean Reads” are saved in FASTQ format. HISAT (Hierarchical Indexing for Spliced Alignment of Transcripts) HISAT2 (v2.1.0) [73] software was used for RNA-seq reads to compare reference genomes of *Vitis vinifera* (http://plants.ensembl.org/Vitis_vinifera/Info/Index?db=core;g=VIT_08s0007g00570;r=8:14828036-14830056;t=VIT_08s0007g00570.t01) (accessed on 10 January 2022). For calculating the mapping rate, Bowtie2 (v2.2.5) was used to align clean reads to the reference gene sequence and then use RSEM to calculate the expression levels of genes and transcripts [74,75]. Successfully mapped clean reads on the reference genome were used for the subsequent bioinformatics analysis.

### 4.4. Quantitative Real-Time PCR (qRT-PCR)

Roche Light Cycler 480 SYBR Green I Master and Roche Light Cycler 480 Real-Time PCR system was used for conducting qRT- PCR. The primers used for the qRT-PCR were shown in Supplementary Table S1 and conditions were as follows: 95 °C for 30 s for denaturation, followed by 40 cycles at 95 °C for 5 s, at 55 °C for 30 s, and at 72 °C for 10 s. Three biological replicates were used for all reactions, and Bio-Rad CFX Manager software was used to determine the threshold cycle (Ct). During the qRT-PCR, method used was as per the manufacturer’s recommendations. The relative quantitative expression level was calculated by the 2^−∆∆CT^ method [76]. The reference gene *VvActin* was used as reference in different grapevine samples tests to analyze gene expression. 

### 4.5. Promoter Isolation of VvPR1 Gene and Sequence Analysis

The genomic DNA was extracted according to the manufacturer’s instructions using DN 15-Plant DNA Mini Kit from the grapevine leaf samples for the isolation of *VvPR1* promoter. A NanoDrop 1000 spectrophotometer (Thermo Scientific, Waltham, MA, USA) was used for measuring the DNA concentration. The promoter of *VvPR1* (VIT_03s0088g00810) gene was isolated by the primer pair (Supplementary Table S2) design on the base of reference sequence of *V. vinifera* L. Approximately 1900 base pair sequence upstream from the coding region supposed to be the putative *VvPR1* promoter. The PCR was performed for the amplification of *VvPR1* promoter by using the Premix high-fidelity (Takara) enzyme. The PCR conditions were: initial denaturation for 2 min at 95 °C, followed by 30 cycles of denaturation for 10 s at 98 °C, annealing for 15 s at 60 °C, and extension for 2 min at 72 °C, and a final extension for 10 min at 72 °C. After the purification of the PCR product on 1.5% agarose gel, it was cloned into the *pCE2* Blunt vector, and sequenced for verification of promoter sequence. To predict the cis-elements in the sequence of *VvPR1*, a promoter online tool PlantCARE was used (http://bioinformatics.psb.ugent.be/webtools/plantcare/html/) (accessed on 17 February 2022) [77]. 

### 4.6. Construction of Beta-Glucuronidase (GUS) Vectors

**Figure 8:**
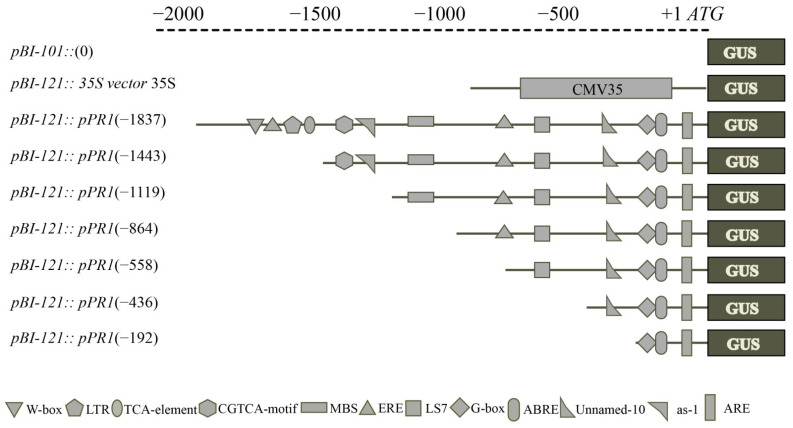
Schematic representation of the *VvPR1* promoter constructs for examining GUS expression in tobacco leaves. The constructs of serial deleted promoter fragments of the *VvPR1* gene were fused to the GUS reporter gene in the vector *pBI-121*.

To construct the vectors of serially deleted *VvPR1* promoter fragments, primers were designed from the sequence of promoter cloned into the *pCE2* Blunt vector (Supplementary Table S2). HindIII restriction site at 5′ end was available on each forward primer and the BamHI restriction site at 5′ end was available on the reverse primer. After the PCR reaction, the purification of the PCR products was done on agarose gel by using the Gel Extraction kit. Meanwhile, the expression vector (*pBI-121*) was also digested by restriction enzymes (HindIII and BamHI) for two hours and then subcloned with the purified PCR products. Seven promoter fragments (−1837 bp to ATG; −1443 bp to ATG; −1119 bp to ATG; −864 bp to ATG; −558 bp to ATG; −436 bp to ATG; and −192 bp to ATG) were separately fused into the expression vector *pBI-121* with the GUS reporter gene, yielding *pBI-121::pPR1* (Figure 8). Expression vector *pBI-121* harboring CaMV35 a strong promoter was used as positive control, and *pBI-101* with no promoter was used as a negative control. DH5α strain of *Escherichia coli* was used to propagate and clone all recombinant plasmid vectors. Then, the constructs of promoter/GUS fusion were inserted into the *Agrobacterium tumefaciens* strain GV3101 by heat shocks.

### 4.7. Agrobacterium-Mediated Transient Expression Assay with Abiotic Stress Treatment

Transient expression assay by *Agrobacterium* was performed according to the method of Yang et al. [78]. *Agrobacterium* GV3101 containing constructs of serially deleted fragments of promoter::GUS were grown on LB medium added with the antibiotics kanamycin (50 µg.mL^−1^) and rifampicin (60 µg.mL^−1^). The strains of *Agrobacterium* were cultured in 50 mL of LB broth at 28 °C overnight. *Agrobacterium* cells were collected from LB broth by the centrifugation at 6000× *g* for 10 min, to adjust an OD600 of 0.8 again resuspended in infiltration media (10 mM MES, 10 mM MgCl_2_, (pH 5.6), 100 µM acetosyringone (Sigma-Aldrich)). The *Agrobacterium* suspension infiltrated into the tobacco leaves by using a needleless syringe; after infiltration, the tobacco plants were placed in a moist and dark chamber at 26 °C for 24 h and then shifted to the growth room. For MeJA treatment, the tobacco leaves harboring the *pBI-121::pPR1*/GUS were treated with 100 µM MeJA (0.2% Tween-20). For control treatment, the tobacco leaves were sprayed with 0.2% Tween-20. Both MeJA-treated and control tobacco plants were packed with polyethylene-perforated plastic bags (with six holes of 1 cm in diameter at each side of 0.03 mm thickness) after putting them into plastic baskets. For wounding treatment, the leaves were picked with a needle. Each treatment was repeated three times with three replications each time on the tobacco leaves for transient GUS expression and samples were collected after 24 h of treatment. 

### 4.8. GUS Activity Measurement

For the qualitative analysis of promoter activity, GUS expression was measured by histochemical staining as explained by Jefferson et al. [79]. GUS staining solution was prepared as mentioned by Yu et al. [80]. Collected tobacco leaf discs were dipped in a GUS staining solution (100 mM NaH_2_PO_4_, 10 mM Na_2_EDTA, 0.5 Mm K_4_Fe(CN)_6_.3H_2_O, 0.5 mg L^−1^ 5-bromo-4-chloro-3- indolyl-b-d-glucuronic acid, and 0.1% Triton X-100 (X-Gluc, Sigma-Aldrich, Shanghai, China), pH 7.0) and incubated for 24 h at 37 °C. For clear observations, chlorophyll contents were removed from leaf discs by incubating in 70% ethanol at 37 °C and rinsed with ethanol several times. In tobacco leaves, quantitative GUS assay of transiently expressed promoter was measured as explained by Jefferson, et al. [80]. Tobacco leaf discs were ground in liquid nitrogen by a mortar and the powder was moved to a microtube. The extraction buffer of one mL (phosphoric acid buffer (2M KPO_4_ (pH 7.8)), 0.5M EDTA, TritonX-100(10%), beta mercapta ethanol, 80% Glycerol) was added and vortexed. The material inside the micro centrifuge tube was centrifuged at 12,000× *g* for 15 min at 4 °C, and the supernatant was transferred to micro centrifuge tube already placed on ice. The whole fluorogenic reaction was performed out in 1 mM 4-methylumbelliferyl-h-d-glucuronide (MUG) (DuchefaBiochemie, Haarlem, The Netherlands) at a volume of 1 mL mixed with extraction buffer, which also contained aliquot of protein extract at volume of 0.1 mL. The quantity of protein extracts were measured by using a standard of bovine serum albumin (BSA) as mentioned by Bradford [81]. At least three times the GUS measurement were repeated with similar results.

## 5. Conclusions

In conclusion, the transcriptional changes in grapevine under the exogenous application of MeJA using RNA-seq technology revealed that dynamic physiological and molecular changes in grapevine leaves occur in response to elicitor application and trigger the plant immune responses. A total of 1242 DEGs were identified; more genes were identified at 48 h of treatment. GO and KEGG analysis revealed that the upregulated DEGs were mainly enriched in MAPK-WRKY pathway, the phytohormone signal transduction pathway jasmonic acid-mediated defense genes, the pathway of flavonoid biosynthesis were all upregulated. The upregulations of these pathways in grapevine leaves help to regulate the ROS, disease-resistance elements, and enhance the hormonal signal transduction in the pathogen’s attack. This transcriptomic study exposed that the defense-related pathways were significantly induced after the exogenous application of MeJA treatment within different time points. Our results validated and expended the available information about MeJA mediated defense responses in grapevine species that are susceptible to various diseases.

## Figures and Tables

**Table 1:** RNA-seq raw data and number of differentially expressed transcripts.

Samples	Total Raw Reads (M)	Total Clean Reads (M)	Clean Reads Q20 (%)	Clean Reads Ratio (%)	Total Mapping (%)	DEGs
CR	44.40	42.46	97.07	95.63	88.17	
MeJA12	43.82	42.59	97.13	97.18	89.01	256
MeJA24	43.82	42.56	97.16	97.12	87.39	294
MeJA48	43.82	42.39	97.14	96.74	86.62	692
Total	175.86	170.00	388.50	386.67	351.19	1242

All transcripts (down- and upregulated) were attained by RNA-seq after treatment with MeJA when compared to controls, according to the Cuffdiff analysis.

**Table 2:** Functional categorization of up- and downregulated genes after treatments with MeJA.

	Name	MeJA 12		MeJA 24		MeJA 48	
		Down	UP	Down	UP	Down	UP
Biological Process	Biological regulation		28	5	35	42	58
	Cellular process		68	14	83	130	225
	Developmental process	1	9	2	10	7	11
	Growth			0	2		
	Immune system process	0	4	0	4	4	9
	Interspecies interaction between organisms	0	5	0	5	1	10
	Localization	7	14	5	13	24	32
	Locomotion	0	3	1	3	9	8
	Metabolic process	14	61	15	81	112	107
	Multi-organism process	0	2	0	2	8	3
	Multicellular organismal process	1	11	1	11	10	13
	Reproduction	0	2	0	2	8	6
	Reproductive process	0	2	0	2	1	13
	Response to stimulus	3	33	9	38	27	57
	Rhythmic process			0	1		
	Signaling	0	18	1	21	18	34
Cellular Component	Cellular anatomical entity			32	131	194	225
	Intracellular			8	56	112	111
	Protein-containing complex			0	8	41	28
Molecular Function	Antioxidant activity			0	5	0	4
	Binding			14	119	113	202
	Catalytic activity			25	127	128	193
	Molecular function regulator			0	5	1	10
	Molecular transducer activity			0	1		
	Protein-folding chaperone			0	1		
	Structural molecule activity			0	3	35	5
	Transcription regulator activity			0	5	6	2
	Translation regulator activity					2	2
	Transporter activity			5	10	16	16

Individual gene products may be assigned to more than one functional category.

**Table 3:** DEGs involved in defense response to MeJA at different time points and transcription factors in DEGs with-fold changes.

Gene Description	Gene ID	Log2 Fold Change		
		MeJA 12	MeJA 24	MeJA 48
PLD	VIT_05s0020g00200	1.37	1.13	1.57
LOX	VIT_01s0010g02750	0.53	0.27	0.88
LOX	VIT_13s0064g01480	2.25	1.90	3.11
ACX	VIT_06s0009g02970	1.57	2.39	1.52
ACX	VIT_06s0009g02880	1.98	3.35	2.88
PAL	VIT_06s0004g02620	2.46	3.27	2.98
CHS	VIT_05s0136g00260	1.70	2.29	2.19
CHS	VIT_14s0068g00920	2.16	2.99	2.77
F3H	VIT_04s0023g03370	3.01	3.95	3.20
F3H	VIT_18s0001g14310	1.03	1.63	1.52
MYB	VIT_15s0046g00170	1.56	2.04	2.59
MYB	VIT_18s0001g09850	0.12	0.82	1.11
WRKY2	VIT_01s0011g00220	0.21	0.27	0.89
WRKY22	VIT_18s0001g10030	0.41	0.62	0.98
PR1	VIT_03s0088g00810	1.64	1.38	2.49
PR4	VIT_03s0088g00780	0.80	0.90	1.12
bHLH	VIT_02s0025g03220	0.40	0.40	1.20

**Table 4:** DEGs involved in MAPK-WRKY pathways in response to MeJA at different time points.

Gene Description	Gene ID	Log2-fold Change		
		MeJA 12	MeJA 24	MeJA 48
FLS2	VIT_09s0002g07680	0.58	1.37	1.70
FLS2	VIT_09s0002g07750	1.32	2.15	2.29
FLS2	VIT_09s0018g00830	0.97	1.04	2.89
FLS2	VIT_09s0054g00100	0.81	2.05	1.99
FLS2	VIT_09s0070g00620	0.25	1.02	1.07
FLS2	VIT_11s0118g00160	1.50	2.37	2.02
FLS2	VIT_13s0067g00420	3.01	3.40	3.21
EFR	VIT_08s0007g00850	2.61	2.16	2.89
EFR	VIT_18s0166g00050	2.41	2.60	3.39
BAK1	BGI_novel_G000939	2.54	4.98	4.72
BRI1	VIT_07s0031g01850	0.67	0.60	0.94
MEKK1	VIT_12s0142g00700	3.54	2.06	3.99

## Data Availability

The datasets are available at NCBI, with BioSample accession PRJNA845078; from SAMN28854480 to SAMN28854491 every treatment three replicates; and the BioProject’s metadata are available at https://www.ncbi.nlm.nih.gov/search/all/?term=PRJNA845078 accessed on 3 May 2022.

## Supplementary Materials

The following supporting information can be downloaded at: https://www.mdpi.com/article/10.3390/plants11121540/s1, Figure S1: Kyoto Encyclopedia of Genes and Genomes (KEGG) enrichment analysis of specifically differentially expressed genes (DEGs) at 12, 24, 48 h of MeJA treatment; Table S1: Primers list used for qRT-PCR; Table S2: List of primers used for cloning and serial deletion of promoter; Table S3: DEGs involved in defence response to MeJA at different time points and transcription factors in DEGs FPKM values; Table S4: Predicted cis-elements in *VvPR1* promoter sequence by PlantCARE promoter databases.
