# Supplementary material for: The Promoter Analysis of VvPR1 Gene: A Candidate Gene Identified through Transcriptional Profiling of Methyl Jasmonate Treated Grapevine (Vitis vinifera L.)
Source: Plants (Basel). 2022 Jun 9;11(12):1540. doi: 10.3390/plants11121540 (PMC9227488; doi:10.3390/plants11121540)
Supplement: Supplementary file 1 [file plants-11-01540-s001.zip › plants-1734690-supplementary.pdf]

Supplementry Table S1 Primers list used for qRT-PCR.

Sr #	Name of Primers	Primers	(Forward/Reverse)
1	***VvPAL***	ATGGAATTCTCACACCACAACGG	F
		TCAGCAAATTGGAAGAGGAGTCCC	R
2	***VvCHS***	ACATGGTGGTGGTTGAAGTACCA	F
		CCATTCCAGGCCTTCCCC	R
3	***VvACX***	ACCTCCGGTGTTGACATGC	F
		CAGAACGCCCCATTCCAGG	R
4	***VvF3H***	ATGGCGCCTACGACACTGAC	F
		TTAGGCCAAAATCTGGTCAATGGG	R
5	***VvPR1***	ATGGGGTTGTGTAAGATC	F
		TCAGTAAATAGCAACTATACAAC	R
6	***VvWRKY22***	ATGGCAGTGGAGCTGATGATG	F
		TTAGGATGATTCGAGAACGAGAGCTG	R
7	***VvFLS2***	ATGGAGATGCCAGTCTTTAGCCT	F
		TCACCAGTTGCTTTGAGAAGTGAAG	R
8	***VvBRI1***	ATGGCATGTGAGCTACAGCTC	F
		CTACTGCTTGCTGAATTCCGGG	R
9	***VvMEKK1***	ATGAACTCCAAGCACAAGCA	F
		TTAAGGTCGTATTCCATTAGCCC	R
10	***VvACT1***	CCACCTTGATCTTCATGCTGC	F
		CACCTCCCCATGCTATCCTTCGTC	R

Supplementry Table S2. List of primers used for cloning and serial deletion of promoter

Name of Primers	Primers (Forward/Reverse)
VdPR1	5’TGATAGTCTAGTAGCTGTGAATTTATCCTTGATCAG 3’ F
	5’ TGGAGAGTTCTGGGCACAGC 3’ R
[-1837 to -20]	5’ATGACCATGATTACGCCAAGCTTACCCATCCTTGACATAAGATTTGTCTCAAC 3’F
	5’GACTGACCACCCGGGGATCCTTGATGAGAAGTGAGGCTATTGATGAGAAC 3’R
-1837	5’ACCATGATTACGCCAAAGCTTGATCAGAGTTCTGATTTTGGAGCTTCATGC 3’F
-1443	5’ TATGACCATGATTACGCCAAGCTTCTAAGATTCAATCCTACGTCACTCC 3’ F
-1119	5’ TATGACCATGATTACGCCAAGCTTACTCCAGGCTTTCTCACCAA 3’ F
-864	5’ TATGACCATGATTACGCCAAGCTTTCTTGTAAAGGAGGTTATTGAAACCATA 3’ F
-558	5’TATGACCATGATTACGCCAAGCTTGTGCATTTGAAGATAAGAATCGAAATCA 3’ F
-436	5’TATGACCATGATTACGCCAAGCTTGATATCATACATAGTCTAAAAGGAATCTGAC 3’F
-192	5’ TATGACCATGATTACGCCAAGCTTGCCATTTCATCAGTAGTGTCC 3’ F
R^a	5’ AAGGGACTGACCACCCGGGGATCCGACTCCTACACAACCCCAT 3’ R

R^a is the reverse primer sequence for the deletion fragments except for the (-1837 to -20) primer. Underline below the nucleotide sequence showed the restriction site for restriction enzymes.

Supplementary Table S3 DEGs involved in defence response to MeJA at different time points and transcription factors in DEGs FPKM values

Gene Description	Gene ID	FPKM values			
		Control	MeJA 12	MeJA 24	MeJA 48
PLD	VIT_05s0020g00200	6.73	17.51	14.63	16.77
LOX	VIT_01s0010g02750	10.39	16.44	13.43	17.59
LOX	VIT_13s0064g01480	0.03	1.52	0.69	0.54
ACX	VIT_06s0009g02970	2.96	10.86	20.32	8.61
ACX	VIT_06s0009g02880	0.16	2.44	5.36	2.85
PAL	VIT_06s0004g02620	3.48	32.73	53.44	34.87
CHS	VIT_05s0136g00260	62.80	255.97	380.29	295.91
CHS	VIT_14s0068g00920	15.78	97.84	185.41	122.76
F3H	VIT_04s0023g03370	2.88	46.63	89.47	32.49
F3H	VIT_18s0001g14310	37.32	90.39	140.48	105.57
MYB	VIT_15s0046g00170	2.71	10.61	15.48	22.84
MYB	VIT_18s0001g09850	73.66	87.42	145.17	156.89
WRKY2	VIT_01s0011g00220	12.47	15.38	16.23	21.63
WRKY22	VIT_18s0001g10030	15.91	22.90	27.02	29.44
PR1	VIT_03s0088g00810	51.50	220.50	195.28	403.63
PR4	VIT_03s0088g00780	3350.30	5800.29	6264.61	7316.73
bHLH	VIT_02s0025g03220	17.40	25.41	24.95	37.55

Supplementary Table S4 Predicted cis-elements in *VvPR1* promoter sequence by PlantCARE and PLACE promoter databases

Motif	Copies	Sequence	Function
TATA-box	42	TATA<br>TATATA<br>ATATAT	Core promoter element
ABRE	1	ACGTG	Abscisic acid responsiveness Cis- element
CGTCA-motif	2	CGTCA	MeJA-responsiveness Cis- element
ERE	2	ATTTTAAA	Ethylene responsive
G-box	1	TACGTG	Cis-acting regulatory element involved in light responsiveness
LS7	1	CAGATTTATTTTTA	Part of a light responsive element
MBS	2	CAACTG	MYB binding site involved in drought-inducibility
ARE	3	AAACCA	Cis- element essential for the anaerobic induction
AT1-motif	2	AATTATTTTTTATT	Part of a light responsive module
CAAT-box	29	CAAT	Common cis- element in promoter and enhancer regions

		CCAAT	
		CAAAT	
GATA-motif		AAGATAAGATT	Cis- element required for high level light regulated and tissue specific
TCA-element	1	CCATCTTTTT	Cis-acting element involved in salicylic acid responsiveness
W box	1	TTGACC	Defense related (recognized especially by Salicylic acid (SA)-induced WRKY DNA binding protein)
Circadian	1	CAAAGATATC	Cis- element involved in circadian control
as-1	1	TGACG	Binding site for TGA transcription factors
LTR	1	CCGAAA	Cis- involved in low-temperature responsiveness
MYC	3	CAATTG	MYC recognition elements
STRE	2	AGGGG	Stress responsive element
Circadian	1	CAAAGATATC	Cis- element involved in circadian control

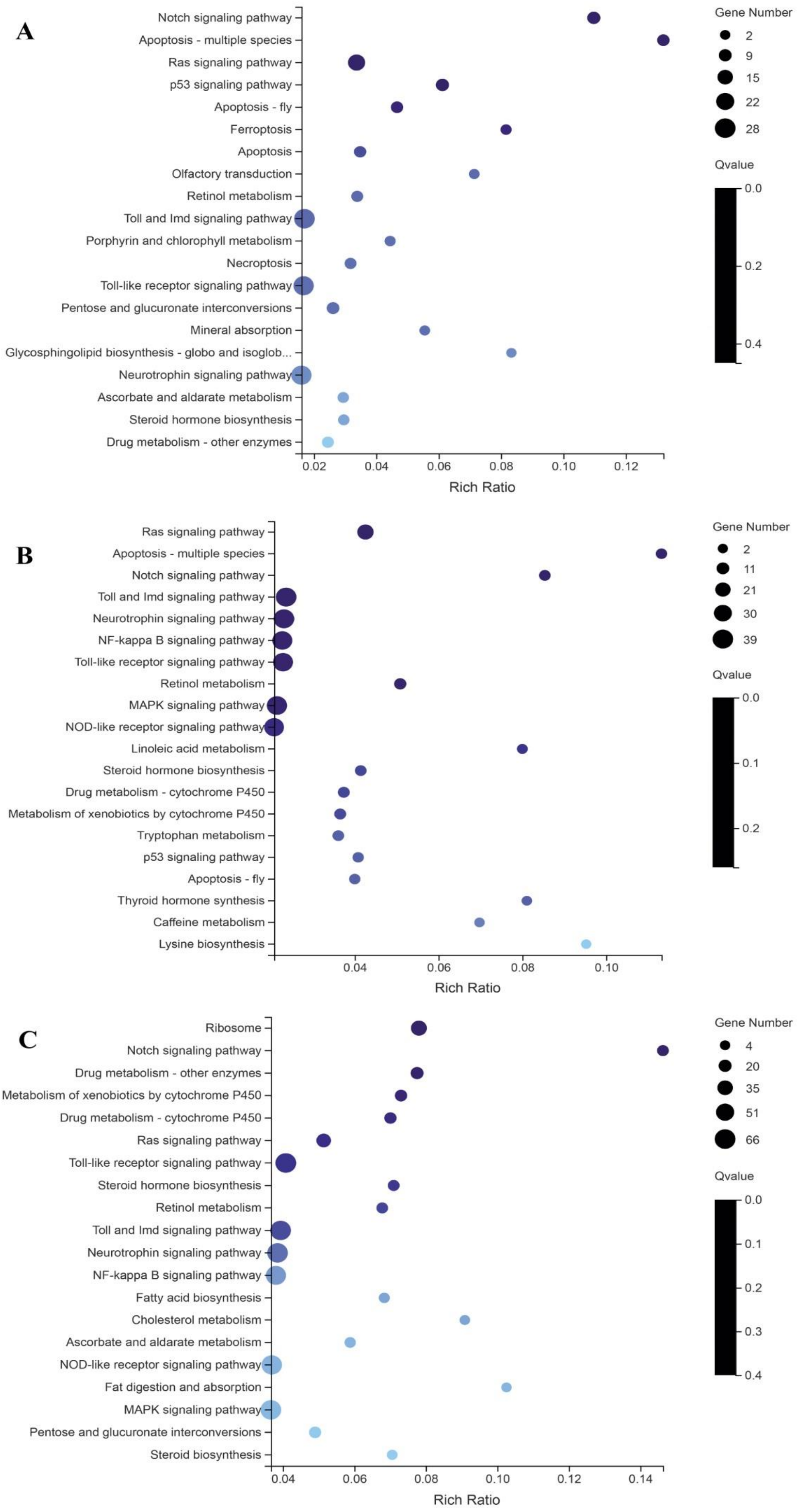

A
Notch signaling pathway
Apoptosis - multiple species
Ras signaling pathway
p53 signaling pathway
Apoptosis - fly
Ferroptosis
Apoptosis
Olfactory transduction
Retinol metabolism
Toll and Imd signaling pathway
Porphyrin and chlorophyll metabolism
Necroptosis
Toll-like receptor signaling pathway
Pentose and glucuronate interconversions
Mineral absorption
Glycosphingolipid biosynthesis - globo and isoglob...
Neurotrophin signaling pathway
Ascorbate and aldarate metabolism
Steroid hormone biosynthesis
Drug metabolism - other enzymes
0.02
0.04
0.06
0.08
0.10
0.12
Rich Ratio
Gene Number
2
9
15
22
28
Qvalue
0.0
0.2
0.4
B
Ras signaling pathway
Apoptosis - multiple species
Notch signaling pathway
Toll and Imd signaling pathway
Neurotrophin signaling pathway
NF-kappa B signaling pathway
Toll-like receptor signaling pathway
Retinol metabolism
MAPK signaling pathway
NOD-like receptor signaling pathway
Linoleic acid metabolism
Steroid hormone biosynthesis
Drug metabolism - cytochrome P450
Metabolism of xenobiotics by cytochrome P450
Tryptophan metabolism
p53 signaling pathway
Apoptosis - fly
Thyroid hormone synthesis
Caffeine metabolism
Lysine biosynthesis
0.04
0.06
0.08
0.10
Rich Ratio
Gene Number
2
11
21
30
39
Qvalue
0.0
0.1
0.2
C
Ribosome
Notch signaling pathway
Drug metabolism - other enzymes
Metabolism of xenobiotics by cytochrome P450
Drug metabolism - cytochrome P450
Ras signaling pathway
Toll-like receptor signaling pathway
Steroid hormone biosynthesis
Retinol metabolism
Toll and Imd signaling pathway
Neurotrophin signaling pathway
NF-kappa B signaling pathway
Fatty acid biosynthesis
Cholesterol metabolism
Ascorbate and aldarate metabolism
NOD-like receptor signaling pathway
Fat digestion and absorption
MAPK signaling pathway
Pentose and glucuronate interconversions
Steroid biosynthesis
0.04
0.06
0.08
0.10
0.12
0.14
Rich Ratio
Gene Number
4
20
35
51
66
Qvalue
0.0
0.1
0.2
0.3
0.4

Supplementary Figure S1 Kyoto Encyclopedia of Genes and Genomes (KEGG) enrichment analysis of specifically differentially expressed genes (DEGs) at 12, 24, 48 h of MeJA treatment. **A**: 12 hour-post treatment, **B:** 24 hour-post treatment, and **C**: 48 hour-post treatment.
